# Addressing advanced HIV disease and mortality in global HIV programming

**DOI:** 10.1186/s12981-020-00296-x

**Published:** 2020-07-10

**Authors:** Andrew T. Boyd, Ikwo Oboho, Heather Paulin, Hammad Ali, Catherine Godfrey, Anand Date, J. Sean Cavanaugh

**Affiliations:** 1 Division of Global HIV and TB, Center for Global Health, Centers for Disease Control and Prevention, 1600 Clifton Road NE, US 1-1, Atlanta, GA 30329 USA; 2 Office of the Global AIDS Coordinator, Washington, DC USA

**Keywords:** HIV, Advanced HIV disease, Mortality, PEPFAR, Opportunistic infections, Tuberculosis

## Abstract

**Introduction:**

The US President’s Emergency Plan for AIDS Relief (PEPFAR) was launched to increase access to antiretroviral treatment (ART) among people living with HIV (PLHIV) and to prevent new HIV infections globally. As new infections have decreased in many PEPFAR-supported countries, PEPFAR is increasingly focusing on understanding and decreasing mortality among PLHIV, specifically by addressing advanced HIV disease (AHD) and its attendant opportunistic infections (OIs). Several developments in identifying AHD, in preventing, diagnosing, and treating selected OIs, and in PEPFAR’s support for mortality surveillance make this an opportune moment for PEPFAR to address HIV-related mortality.

**Discussion:**

AHD upon diagnosis or re-engagement in HIV care is not uncommon, and it substantially increases risk of death from OIs. The World Health Organization provides evidence-based guidelines for a package of interventions for preventing, diagnosing, and treating common OIs, including tuberculosis (TB), cryptococcal meningitis, and severe bacterial infections. PEPFAR facilitates implementation of these guidelines. To identify PLHIV with low CD4, PEPFAR plans to support expanded access to CD4 testing, including a point-of-care assay that differentiates CD4 cell count as a binary of greater than or less than 200 cells/µL. To prevent AHD-related mortality, PEPFAR supports rapid ART initiation with integrase inhibitor–based regimens and implementation and documentation of TB preventive treatment. To diagnose selected OIs, PEPFAR is implementing urine lateral flow lipoarabinomannan use to identify TB among PLHIV who have a CD4 cell count < 200 cells/µL. To treat selected OIs, PEPFAR has focused on improving patient-centered care in TB/HIV co-infection services and scaling up implementation of new drug regimens for cryptococcal meningitis. To better understand mortality, PEPFAR has introduced an indicator, TX_ML, to routinely and systematically categorize outcomes, including deaths, among PLHIV on ART.

**Conclusions:**

PEPFAR is increasing its efforts to identify AHD; to prevent, diagnose, and treat OIs; and to track mortality in its programs. These ongoing efforts, done in collaboration with other stakeholders, seek to decrease mortality among PLHIV.

## Introduction

The President’s Emergency Plan for AIDS Relief (PEPFAR), launched by the United States government in 2003, was initially conceived to increase access to lifesaving antiretroviral treatment (ART) among people living with HIV (PLHIV) in low-income countries and to prevent new HIV infections. PEPFAR’s reach continues to expand: as of September 2019, PEPFAR was sustaining nearly 15.7 million PLHIV on treatment [1].

PEPFAR’s goal is to achieve HIV epidemic control, defined as the point at which the number of new HIV infections is less than the number of all-cause deaths among PLHIV, and when both infections and deaths are decreasing, in the countries it supports [2, 3]. To date, PEPFAR has focused on decreasing new HIV infections through prevention, case-finding, and treatment efforts, and in most countries, new infections are indeed decreasing [4, 5]. HIV-related mortality also has declined globally, by about one-third between 2010 and 2018; however, there were still 770,000 HIV-related deaths (uncertainty bounds: 570,000–1.1 million) in 2018 [5]. Realizing a meaningful vision of epidemic control means that PEPFAR and HIV program implementers must work not only to drive down infections, but also mortality.

To that end, PEPFAR has become increasingly engaged in understanding and addressing mortality among PLHIV, specifically by addressing advanced HIV disease (AHD) with opportunistic infections (OIs). This commentary reviews the epidemiology of AHD and describes recent developments in identifying AHD and in preventing, diagnosing, and treating selected OIs. It describes the ways in which PEPFAR and other global HIV program stakeholders are applying these developments to decrease mortality among PLHIV. Finally, it discusses PEPFAR’s current efforts in supporting surveillance of mortality among PLHIV to guide programmatic priorities in decreasing mortality in this population.

## Epidemiology of advanced HIV disease and mortality among PLHIV

HIV-related mortality is higher among PLHIV who present to or re-enter clinical care with AHD than among those PLHIV who do not present with AHD [6, 7]. Among all PLHIV aged ≥ 5 years, AHD is defined as having either a CD4 cell count < 200 cells/µL or a World Health Organization (WHO) HIV clinical stage 3 or 4 condition at presentation for care; all children living with HIV aged < 5 years are categorized as having AHD [4, 8]. AHD occurs among both ART-naïve and ART-experienced individuals. The most common causes of death among adults with AHD are tuberculosis (TB), cryptococcal meningitis, and severe bacterial infections (SBIs) [4, 9, 10].

Although estimates of AHD prevalence among PLHIV vary, AHD is not uncommon. Data from South Africa show nearly one-third of HIV patients entering care have AHD [11]. Preliminary results from community-based Population HIV Impact Assessment surveys conducted during 2016–2018 in nine PEPFAR-supported countries found 11─22% of newly-diagnosed adult PLHIV had a CD4 cell count < 200 cells/µL [12].

Identifying AHD is crucial to ensure that these PLHIV receive focused interventions against associated OIs. Early intervention is key, because AHD-related death can occur soon after initial presentation or re-engagement in care: among the REALITY trial cohort of PLHIV with CD4 cell count < 100 cells/µL, 12.7% died within 48 weeks of enrollment, with the highest mortality occurring during the first 4 weeks on ART [13]. To combat AHD-related mortality, WHO provides guidelines for timely application of a package of care [4]. The package includes interventions for preventing, diagnosing, and treating common OIs, including TB, cryptococcal meningitis, and SBIs, and for ensuring rapid initiation on ART and intensified adherence support.

TB is the most common identified cause of death among PLHIV worldwide, accounting for one-third of all HIV-related deaths in 2018 [14], but its role in mortality is likely underestimated. A 2015 meta-analysis of 36 autopsy studies among PLHIV in resource-limited settings found that TB was considered the cause of death in 91.4% (95% CI 85.8–97.0%) of those with TB. Additionally, TB remained undiagnosed at death in 45.8% (95% CI 32.6–59.1%) of TB cases [15]. PLHIV may have limited symptoms of pulmonary TB or unrecognized extrapulmonary TB. Furthermore, because TB incidence among PLHIV on ART remains up to six times higher than among those not infected with HIV, adjunctive strategies to reduce TB-related mortality are critical. For instance, treating TB infection among PLHIV, after controlling for ART, was associated with a 39% reduction in mortality [16].

Cryptococcal meningitis accounts for 15% of acquired immunodeficiency syndrome (AIDS)-related deaths and 15–20% of deaths among hospitalized PLHIV [9, 17]. Cryptococcal antigen (CrAg) can be detected in the blood before the development of meningitis, and cryptococcal antigenemia is seen more frequently among PLHIV with CD4 cell count < 100 cells/µL than among PLHIV with higher CD4 cell counts [17]. Recent data show that cryptococcal antigenemia is also common in PLHIV with CD4 cell count < 200 cells/µL [18]. Treatment for cryptococcal meningitis is labor-intensive and difficult in low-income countries.

SBIs comprise major organ system infections including bacterial pneumonia, isolated bacteremia, and severe diarrhea. They are estimated to contribute to over one-third of hospitalizations among PLHIV globally and are a leading cause of death [9]. In the REALITY trial, among PLHIV with CD4 cell count < 100 cells/µL, the most common cause of death was “unknown” (5% cumulative incidence), while confirmed SBI accounted for 1.9%. However, it is hypothesized that some of the burden of unknown cause of death might be attributed to undiagnosed SBIs [13, 19]. Although these infections are not universally considered OIs, incidence is lower in people who start ART immediately and maintain immune function [20, 21]. If diagnosed or suspected, these can generally be treated with conventional antibiotics.

Despite the WHO guidelines to prevent AHD-related mortality among PLHIV, there are significant logistical and financial barriers to identifying PLHIV with AHD and implementing these interventions. PEPFAR and HIV program implementers play a significant role in facilitating guidelines implementation by helping clinicians identify those with AHD and by expanding access to evidence-based innovations in preventing, diagnosing, and treating OIs. A summary of how PEPFAR supports these interventions can be found in Table 1.

**Table 1:** Summary of PEPFAR supports for interventions to identify advanced HIV disease; prevent, diagnose, and treat opportunistic infections; and conduct surveillance of mortality among PLHIV

Category	Intervention	How PEPFAR supports the intervention
Identifying advanced HIV disease (AHD)	Use of a point-of-care test that differentiates CD4 cell count as binary greater than or less than 200 cells/µL	Once test is WHO pre-qualified, PEPFAR plans to support use of this test to target PLHIV at increased risk of AHD
Preventing AHD-related mortality and associated opportunistic infections (OIs)	Rapid ART initiation using optimized ART	Rapid ART initiation as cornerstone of HIV programming, scaling up integrase-based ART regimens
	TB preventive treatment	Commitment to ensure all eligible PLHIV on ART receive TPT by 2022
	Cryptococcal antigen (CrAg) testing and cryptococcal meningitis preventive interventions	Recognition of importance of CrAg testing and cryptococcal meningitis preventive interventions in PEPFAR guidance
	Prevention of severe bacterial infections (SBI) using co-trimoxazole	Co-trimoxazole is mainstay of PEPFAR guidance and programming
Diagnosing OIs	Use of Xpert MTB/RIF testing among PLHIV with presumptive TB	Improving implementation of Xpert MTB/RIF, through optimizing distribution of machines, improving specimen transfer networks, and scaling up Xpert MTB/RIF Ultra
	Use of urine lateral flow lipoarabinomannan (LF-LAM) to diagnose and screen for active TB among PLHIV with AHD^1^	PEPFAR guidelines recommend use of LF-LAM among PLHIV with a CD4 cell count < 200 cells/µL
Treating OIs	Integration of HIV and TB services	PEPFAR programming emphasizes co-location of HIV and TB services and timely return of TB testing results to inform ART initiation
	Option of all-oral induction therapy for cryptococcal meningitis	Working with other global HIV programmers in scaling up clinical implementation of this all-oral option
Surveillance of mortality among PLHIV	Conduct systematic surveillance of mortality	Use of an indicator TX_ML in its data reporting systems to track loss to follow up, including loss from mortality, allowing routine reporting on mortality

^1^WHO 2019 guidelines for the use of LF-LAM in the diagnosis of active TB among PLHIV include the following: among inpatients with signs and symptoms of TB, with AHD, or with CD4 cell count < 200 cells/μL; and among outpatients with signs and symptoms of TB or with CD4 cell count < 100 cells/μL

## Identifying AHD, and preventing, diagnosing, and treating selected OIs

### Identifying AHD

The first component in ensuring persons with AHD receive the care they need is to identify AHD. Relying on WHO clinical staging alone to identify AHD misses a significant number of PLHIV with CD4 cell count < 200 cells/µL [22], and almost half of those with CD4 cell count < 100 cells/µL, according to the REALITY trial [23]. In PEPFAR-supported country programs, when the policy for ART initiation criteria shifted from CD4 cell count thresholds to treatment for all, routine CD4 testing was de-emphasized to use resources to scale up viral load testing. Currently, PEPFAR supports limited CD4 testing among targeted groups receiving AHD services in HIV service delivery facilities, among PLHIV at presentation in areas with high AHD prevalence, or among individuals aged ≥ 5 years who have documented viremia despite receiving ART for more than a year [1, 12]. The challenge with this current approach is that persons who are not initially identified by clinical staging may never be identified as having AHD, and thus would miss AHD services. An inexpensive, reliable, point-of-care (POC) assay that allows identification of AHD would help tailor care for PLHIV with AHD.

To that end, a POC test has been developed that differentiates the CD4 cell count as a binary greater than or less than 200 cells/µL, and it is undergoing WHO pre-qualification for use. By rapidly identifying PLHIV with lower CD4 count, the test can help determine who needs screening and prophylaxis for OIs. It also indicates who should have intensified AHD-focused differentiated service delivery. Once the test is WHO pre-qualified, and with necessary training for those conducting the test, PEPFAR plans to support use of this test to target PLHIV at increased risk of AHD at entry or re-entry into care [1, 12].

### Preventing AHD-related mortality and associated OIs

ART is the most important intervention to prevent AHD-related mortality. Rapid ART initiation or re-initiation with regimens that rapidly reduce viral load with few adverse events, such as integrase inhibitor-based regimens, is critical [24, 25]. Although rapidly decreasing viral load with integrase inhibitor-based regimens could increase risk of immune reconstitution inflammatory syndrome, data on this risk and its consequences are mixed [26], and use of these drugs is scaling up in PEPFAR programs. Finally, the implementation of index case testing among newly-identified PLHIV to seek and bring to care other PLHIV also facilitates early diagnosis of PLHIV. Rapid ART initiation, age-specific ART optimization, and index case testing are all cornerstones of PEPFAR policy.

Another PEPFAR priority is TB preventive treatment (TPT) for PLHIV, as several developments have made scale-up of TPT among PLHIV plausible. Shorter TPT regimens, with fewer serious hepatotoxic adverse events and improved adherence compared with isoniazid alone for 6 months, have been recommended by WHO for use among PLHIV [27, 28]. Political will for global TPT scale-up is increasing: the 2018 United Nations High-Level Meeting on TB pledged to ensure that 6 million PLHIV receive TPT by 2022, with PEPFAR committing to ensuring that all eligible PLHIV on ART receive TPT. Finally, PEPFAR is working to improve TPT tracking and course completion.

PEPFAR guidance recognizes that CD4-directed CrAg testing and preventive interventions may improve outcomes in those with AHD [12]. The REMSTART study demonstrated reduced mortality among PLHIV presenting with CD4 cell count < 200 cells/µL who received serum CrAg screening, pre-emptive antifungal therapy with fluconazole for those screening CrAg positive, and intensified treatment support for the first month of ART [29]. Some experts contend that adding flucytosine to standard pre-emptive fluconazole therapy for 2 weeks may help prevent progression to cryptococcal meningitis among PLHIV screening CrAg-positive [30]. However, this potential combination therapy is not yet part of WHO guidelines or PEPFAR guidance.

Combination therapies also have been investigated to prevent SBIs among PLHIV with AHD. In the REALITY trial, a cohort of PLHIV with CD4 cell count < 100 cells/µL received a package of enhanced prophylaxis (ART, continuous co-trimoxazole, 12 weeks of isoniazid/pyridoxine, 12 weeks of fluconazole in the absence of CrAg testing, 5 days azithromycin, and a single dose of albendazole), while the control group received ART and co-trimoxazole alone; the enhanced prophylaxis group had lower mortality rates than the control group at 24 and 48 weeks [23]. However, bacterial infection-related mortality rates did not differ between the groups. Further studies assessing the role of antibiotics in preventing mortality from SBIs among PLHIV with AHD are needed. Currently, co-trimoxazole alone remains a mainstay of WHO guidelines and PEPFAR guidance for preventing SBIs.

### Diagnosing selected OIs associated with AHD

Xpert MTB/RIF testing among PLHIV with presumptive TB is part of WHO guidelines, and PEPFAR has worked to improve its implementation. This work includes optimizing distribution of GeneXpert machines, utilizing full machine capacity, improving specimen and result transfer networks, and scaling up the more sensitive Xpert MTB/RIF Ultra cartridge [31]. WHO guidelines also recommend using urine lateral flow lipoarabinomannan (LF-LAM) to diagnose and screen for active TB among PLHIV as follows: among inpatients with signs and symptoms of TB, with AHD, or with CD4 cell count < 200 cells/µL; and among outpatients with signs and symptoms of TB or with CD4 cell count < 100 cells/µL [4, 32]. A new urine LF-LAM test, with improved sensitivity and preserved specificity in comparison with the currently used LF-LAM test, may soon be available and is expected to improve the diagnosis of TB among PLHIV with AHD [33]. However, much work remains to be done in scaling up the implementation of LF-LAM among PLHIV with AHD or severe illness. In alignment with Médecins Sans Frontières recommendations and in anticipation of the programmatic use of the POC CD4 cell count test, PEPFAR guidelines recommend use of LF-LAM among PLHIV with a CD4 cell count < 200 cells/µL.

The use of molecular POC (or near POC) testing for TB is well-established, but its use in diagnosing SBIs is still somewhat premature. However, there are emerging data on the use of molecular diagnostic arrays for patients, including PLHIV, who are severely ill [34–36]. Developing microarray- and cartridge-based technologies for diagnosis of SBIs may guide timely, pathogen-specific interventions among PLHIV with AHD.

### Treating selected OIs associated with AHD

In managing TB in PLHIV, simplifying TB treatment regimens, including for drug-resistant TB, has been a key development. Current evidence-based recommendations include all-oral regimens that incorporate bedaquiline and linezolid for rifampin-resistant TB, and mounting evidence supports using three oral drugs to treat all forms of drug-resistant TB [37]. Improved integration of HIV and TB services, including co-location of services and timely return of TB testing results before ART initiation, is an important component of PEPFAR programming. Additionally, recent emphasis on patient-centered care in PEPFAR programming has led to rethinking the paradigm of directly-observed therapy (DOT) for TB treatment, including relaxation of DOT requirements or introducing new forms of DOT, such as video-assisted treatment or text message confirmation.

In managing cryptococcal meningitis, a major update in the 2018 WHO guidelines was a recommendation of an all-oral induction regimen of 2 weeks of fluconazole and flucytosine as an alternative option to amphotericin B and flucytosine. This update was informed by the ACTA trial, which demonstrated that 1  week of intravenous amphotericin B plus oral flucytosine followed by 1 week of oral fluconazole, and 2 weeks of oral fluconazole plus flucytosine, were non-inferior to the previous gold standard of amphotericin for 2 weeks of induction therapy for cryptococcal meningitis [38]. The use of an all-oral induction regimen decreases cost of treatment and requires no exposure to amphotericin, a relatively toxic drug. Flucytosine, while also expensive, has fewer associated side effects than amphotericin, and so may be attractive for programmatic use. As a result, UNITAID is working to drive down costs of these drugs while working with partners, including Global Fund and PEPFAR, to scale up their clinical implementation.

Pathogen-directed treatment of SBIs depends on improvement in diagnosis, but algorithmic approaches to the presumptive treatment of such infections remain a critical area of study [39, 40].

## Surveillance of mortality among PLHIV on ART in PEPFAR programs

To understand mortality among PLHIV on ART it serves, PEPFAR supports several approaches to mortality surveillance, including civil registration and vital statistics (CRVS) systems, verbal autopsy, and mortuary surveillance. However, these approaches are not standardized across all PEPFAR country programs and lack reporting requirements, limiting the utility of these data. To address these limitations, in October 2018 PEPFAR introduced a new indicator, TX_ML, defined as the number of PLHIV on ART with no clinical contact after 28 days since their last expected contact, in its data reporting systems focused on loss to follow-up (LTFU), including LTFU from mortality, among all PLHIV on ART supported by PEPFAR [41, 42].

TX_ML reporting is intended to ensure programmatic action is being taken to locate PLHIV who have missed clinical visits, and that outcomes are accurately documented. [12]. It is the first PEPFAR indicator to systematically categorize outcomes among those reported as LTFU, and requires reporting on the number of PLHIV who died, who were not traced or were unsuccessfully traced (the truly LTFU), who transferred out, or who stopped ART [42].

Implementation of this indicator marks the first time that PEPFAR has required routine reporting on mortality among PLHIV on ART. This indicator also includes optional reporting on cause of death (COD). In the absence of robust CRVS systems, PEPFAR has also provided guidance on the method of standardized mortality recording and reporting, by recording in-facility deaths in a dedicated tracking register and utilizing standard Medical Certificate of Death and COD forms. For deaths in the community, verbal autopsy is recommended.

These data are collected not only for surveillance purposes but also to inform programmatic activity. Mortality data can be compared between sites and districts, as well as by age and sex, to determine the geographic areas and demographic groups with higher proportions of deaths or types of deaths, thus guiding specific interventions. Tracking mortality data over time can reveal important epidemiologic trends, including the number of all-cause deaths among PLHIV. PEPFAR guidance encourages developing or strengthening national CRVS systems, which can then be used to track other disease-specific mortality or to assess the impact of public health interventions. Additionally, this focus on mortality as an outcome is a key component of strengthening HIV case-based surveillance, a cornerstone of sustainable HIV programs. Ultimately, documenting COD among PLHIV in PEPFAR programs can focus resources on interventions aimed at decreasing preventable deaths.

## Conclusions

The mandates of HIV programming are to prevent the spread of infection and reduce morbidity and mortality associated with HIV. PEPFAR and other global HIV stakeholders, through a focus on HIV prevention, case-finding, and provision of ART, have made a substantial impact in decreasing new HIV infections in low-income countries. Comprehensively addressing HIV-related mortality incorporates identifying and treating PLHIV with AHD; preventing, diagnosing, and treating OIs; tracing PLHIV lost from care; and improving mortality surveillance. These efforts include expanding access to integrase inhibitor-based ART regimens, diagnostics distinguishing between PLHIV with low CD4 and higher CD4 cell counts, and differentiated, intensified care for PLHIV with AHD. These efforts are necessarily collaborative, with PEPFAR working with Ministries of Health and international organizations to advance this approach.

The current COVID-19 pandemic has introduced a new threat to the care and well-being of PLHIV. Though research to date is not definitive, PLHIV may suffer a higher rate of severe disease from COVID itself [43, 44] and, perhaps more importantly, from discontinuity of HIV and co-morbidity care while health services are limited or shifted to COVID response. PEPFAR is working to adapt its service delivery models to both mitigate risk of COVID transmission among PLHIV and to maintain continuity of HIV care; these adaptations include an acceleration in multi-month dispensing to minimize need for clinic visits, development of true community care models, and changes in clinic flow and use of space to minimize risk to PLHIV in facilities. In this way, PEPFAR and other global HIV programmers can continue to reduce mortality among PLHIV.

## Data Availability

Not applicable.

## References

[1] PEPFAR. PEPFAR 2020 Country Operational Plan Guidance for all PEPFAR Countries. 2019.

[2] PEPFAR 3.0: Controlling the epidemic: Delivering on the promise of an AIDS-free generation [press release]. 2014.

[3] PEPFAR Strategy for accelerating HIV/AIDS epidemic control (2017–2020) [press release]. 2017.

[4] World Health Organization (2017). Guidelines for managing advanced HIV disease and rapid initiation of antiretroviral therapy, July 2017.

[5] Global HIV & AIDS statistics 2019 fact sheet [press release]. 2019.

[6] Egger M, May M, Chene G, Phillips AN, Ledergerber B, Dabis F (2002). Prognosis of HIV-1-infected patients starting highly active antiretroviral therapy: a collaborative analysis of prospective studies. Lancet.

[7] Walker AS, Prendergast AJ, Mugyenyi P, Munderi P, Hakim J, Kekitiinwa A (2012). Mortality in the year following antiretroviral therapy initiation in HIV-infected adults and children in Uganda and Zimbabwe. Clin Infect Dis.

[8] Waldrop G, Doherty M, Vitoria M, Ford N (2016). Stable patients and patients with advanced disease: consensus definitions to support sustained scale up of antiretroviral therapy. Trop Med Int Health.

[9] Ford N, Shubber Z, Meintjes G, Grinsztejn B, Eholie S, Mills EJ (2015). Causes of hospital admission among people living with HIV worldwide: a systematic review and meta-analysis. Lancet HIV..

[10] Ford N, Doherty M (2017). The enduring challenge of advanced HIV infection. N Engl J Med.

[11] Carmona S, Bor J, Nattey C, Maughan-Brown B, Maskew M, Fox MP (2018). Persistent high burden of advanced HIV disease among patients seeking care in South Africa’s National HIV Program: data from a Nationwide Laboratory Cohort. Clin Infect Dis..

[12] PEPFAR. PEPFAR 2019 Country operational plan guidance for all PEPFAR Countries. 2019.

[13] Post FA, Szubert AJ, Prendergast AJ, Johnston V, Lyall H, Fitzgerald F (2018). Causes and timing of mortality and morbidity among late presenters starting antiretroviral therapy in the REALITY Trial. Clin Infect Dis.

[14] World Health Organization (2019). Global tuberculosis report 2019.

[15] Gupta RK, Lucas SB, Fielding KL, Lawn SD (2015). Prevalence of tuberculosis in post-mortem studies of HIV-infected adults and children in resource-limited settings: a systematic review and meta-analysis. AIDS..

[16] Badje A, Moh R, Gabillard D, Guehi C, Kabran M, Ntakpe JB (2017). Effect of isoniazid preventive therapy on risk of death in west African, HIV-infected adults with high CD4 cell counts: long-term follow-up of the Temprano ANRS 12136 trial. Lancet Glob Health..

[17] Rajasingham R, Smith RM, Park BJ, Jarvis JN, Govender NP, Chiller TM (2017). Global burden of disease of HIV-associated cryptococcal meningitis: an updated analysis. Lancet Infect Dis..

[18] Ford N, Shubber Z, Jarvis JN, Chiller T, Greene G, Migone C (2018). CD4 cell count threshold for cryptococcal antigen screening of HIV-infected individuals: a systematic review and meta-analysis. Clin Infect Dis.

[19] Prabhu S, Harwell JI, Kumarasamy N (2019). Advanced HIV: diagnosis, treatment, and prevention. Lancet HIV..

[20] O’Connor J, Vjecha MJ, Phillips AN, Angus B, Cooper D, Grinsztejn B (2017). Effect of immediate initiation of antiretroviral therapy on risk of severe bacterial infections in HIV-positive people with CD4 cell counts of more than 500 cells per muL: secondary outcome results from a randomised controlled trial. Lancet HIV..

[21] Anglaret X, Eholie SP (2017). Severe bacterial infections: overlooked opportunistic diseases. Lancet HIV..

[22] Munthali C, Taegtmeyer M, Garner PG, Lalloo DG, Squire SB, Corbett EL (2014). Diagnostic accuracy of the WHO clinical staging system for defining eligibility for ART in sub-Saharan Africa: a systematic review and meta-analysis. J Int AIDS Soc..

[23] Hakim J, Musiime V, Szubert AJ, Mallewa J, Siika A, Agutu C (2017). Enhanced prophylaxis plus antiretroviral therapy for advanced HIV infection in Africa. N Engl J Med.

[24] Walmsley SL, Antela A, Clumeck N, Duiculescu D, Eberhard A, Gutierrez F (2013). Dolutegravir plus abacavir–lamivudine for the treatment of HIV-1 infection. N Engl J Med.

[25] Clotet B, Feinberg J, van Lunzen J, Khuong-Josses MA, Antinori A, Dumitru I (2014). Once-daily dolutegravir versus darunavir plus ritonavir in antiretroviral-naive adults with HIV-1 infection (FLAMINGO): 48 week results from the randomised open-label phase 3b study. Lancet.

[26] Dutertre M, Cuzin L, Demonchy E, Pugliese P, Joly V, Valantin MA (2017). Initiation of antiretroviral therapy containing integrase inhibitors increases the risk of IRIS requiring hospitalization. J Acquir Immune Defic Syndr.

[27] Sterling TR, Scott NA, Miro JM, Calvet G, La Rosa A, Infante R (2016). Three months of weekly rifapentine and isoniazid for treatment of Mycobacterium tuberculosis infection in HIV-coinfected persons. AIDS..

[28] Swindells S, Ramchandani R, Gupta A, Benson CA, Leon-Cruz J, Mwelase N (2019). One month of rifapentine plus isoniazid to prevent HIV-related tuberculosis. N Engl J Med.

[29] Mfinanga S, Chanda D, Kivuyo SL, Guinness L, Bottomley C, Simms V (2015). Cryptococcal meningitis screening and community-based early adherence support in people with advanced HIV infection starting antiretroviral therapy in Tanzania and Zambia: an open-label, randomised controlled trial. Lancet.

[30] Loyse A, Burry J, Cohn J, Ford N, Chiller T, Ribeiro I (2019). Leave no one behind: response to new evidence and guidelines for the management of cryptococcal meningitis in low-income and middle-income countries. Lancet Infect Dis..

[31] Pathmanathan I, Date A, Coggin WL, Nkengasong J, Piatek AS, Alexander H (2017). Rolling out Xpert((R)) MTB/RIF for TB detection in HIV-infected populations: an opportunity for systems strengthening. Afr J Lab Med..

[32] World Health Organization (2019). Lateral flow urine lipoarabinomannan assay (LF-LAM) for the diagnosis of active tuberculosis in people living with HIV, Policy update (2019).

[33] Broger T, Sossen B, du Toit E, Kerkhoff AD, Schutz C, Ivanova Reipold E (2019). Novel lipoarabinomannan point-of-care tuberculosis test for people with HIV: a diagnostic accuracy study. Lancet Infect Dis..

[34] Moore CC, Jacob ST, Banura P, Zhang J, Stroup S, Boulware DR (2019). Etiology of sepsis in Uganda using a quantitative polymerase chain reaction-based TaqMan array card. Clin Infect Dis..

[35] Siddiqi OK, Ghebremichael M, Dang X, Atadzhanov M, Kaonga P, Khoury MN (2014). Molecular diagnosis of central nervous system opportunistic infections in HIV-infected Zambian adults. Clin Infect Dis.

[36] Kozel TR, Burnham-Marusich AR (2017). Point-of-care testing for infectious diseases: past, present, and future. J Clin Microbiol.

[37] WHO (2019). WHO consolidated guidelines on drug-resistant tuberculosis treatment.

[38] Molloy SF, Kanyama C, Heyderman RS, Loyse A, Kouanfack C, Chanda D (2018). Antifungal combinations for treatment of Cryptococcal meningitis in Africa. N Engl J Med.

[39] Zar HJ, Madhi SA, Aston SJ, Gordon SB (2013). Pneumonia in low and middle income countries: progress and challenges. Thorax.

[40] Walusimbi S, Semitala F, Bwanga F, Haile M, De Costa A, Davis L (2016). Outcomes of a clinical diagnostic algorithm for management of ambulatory smear and Xpert MTB/Rif negative HIV infected patients with presumptive pulmonary TB in Uganda: a prospective study. Pan Afr Med J..

[41] PEPFAR. Monitoring, evaluation, and reporting indicator reference guide (MER 2.0), Version 2.3. 2018.

[42] PEPFAR. Monitoring, evaluation, and reporting indicator reference guide (MER 2.0), Version 2.4. 2019.

[43] Vizcarra P, Perez-Elias MJ, Quereda C, Moreno A, Vivancos MJ, Dronda F (2020). Description of COVID-19 in HIV-infected individuals: a single-centre, prospective cohort. Lancet HIV..

[44] Altuntas Aydin O, Kumbasar Karaosmanoglu H, Kart Yasar K (2020). HIV/SARS-CoV-2 coinfected patients in Istanbul. J Med Virol..

